# Development and Applications of Prognostic Risk Models in the Management of Invasive Mold Disease

**DOI:** 10.3390/jof4040141

**Published:** 2018-12-19

**Authors:** Marta Stanzani, Russell E. Lewis

**Affiliations:** 1 Institute of Hematology “Lorenzo e Ariosto Seràgnoli”, Department of Hematology and Clinical Oncology, S. Orsola-Malpighi Hospital, University of Bologna, 40138 Bologna, Italy; marta.stanzani2@unibo.it; 2 Clinic of Infectious Diseases, Department of Medical and Surgical Sciences, S. Orsola-Malpighi Hospital, University of Bologna, 40138 Bologna, Italy

**Keywords:** prognostic risk model, prediction models, risk score, invasive mold disease, hematological malignancy, risk assessment, antifungal stewardship

## Abstract

Prognostic models or risk scores are frequently used to aid individualize risk assessment for diseases with multiple, complex risk factors and diagnostic challenges. However, relatively little attention has been paid to the development of risk models for invasive mold diseases encountered in patients with hematological malignancies, despite a large body of epidemiological research. Herein we review recent studies that have described the development of prognostic models for mold disease, summarize our experience with the development and clinical use of one such model (BOSCORE), and discuss the potential impact of prognostic risk scores for individualized therapy, diagnostic and antifungal stewardship, as well as clinical and epidemiological research.

## 1. Introduction

Many decisions involved in the prevention, diagnosis and treatment of invasive mold disease (IMD) depend on an accurate estimate of the patient’s future risk for developing the infection [1]. This prediction can be challenging given the multivariate and dynamic nature of risk factors that predispose patients to these infections [2,3,4]. Surprisingly little attention has been given to the development of prediction models or risk scores to aid individualized IMD risk assessment, even though such models have been developed for invasive candidiasis in non-neutropenic patients and incorporated in clinical trial design [5,6,7,8]. The concept of developing and validating clinical prediction models for IMD seems logical given that such models have proven to be useful for diseases in other areas of medicine characterized by multivariate and complex risk factors, as well as diagnostic challenges [9,10]. Individualized risk assessment is also a key component of diagnostic and antifungal stewardship efforts, because many evidence-based interventions such as antifungal prophylaxis have only been proven to be clinically beneficial in select high-risk subpopulations [11].

In this review, we will review key risk factors that predispose patients to IMD during the treatment of hematological malignancies, describe recent attempts towards the development prognostic risk models, and explore how these risk models can be incorporated to improve future clinical and epidemiological research. This review is primarily applicable to the development of models for predicting invasive aspergillosis rather than less common molds given the much higher prevalence of this disease, clinical availability of biomarkers for diagnostic-driven management, and evidence-based recommendations for prophylaxis in select patient groups.

## 2. Risk Factors for Invasive Mold Disease

Epidemiological studies performed over the last four decades have identified many risk factors that predispose patients with hematological malignancies to developing an IMD [3]. These include host-specific factors of the (i) type of underlying malignancy and status, whether in remission or active; (ii) Type of immunosuppressive chemotherapy and associated conditions such as neutropenia and damage to integument; (iii) The type of hematopoietic stem cell transplantation (HSCT) (autologous versus allogeneic graft), stem cell source, status of the malignancy at the time of transplant, and genetic risk factors for fungal disease in the donor; (iv) immunosuppressive therapy required to manage graft versus host disease (GVHD); (iv) Patient comorbidities and age; (v) Environmental or occupational risk factors associated with fungal spore exposure; and (vi) A prior history of IMD [3,4].

Although many of these risk factors are discussed in current treatment guidelines [12,13], most diagnostic and treatment algorithms are still generally based on only a few host risk factors used in epidemiological definitions for probable or proven IMD or in drug registration trials [14]. As a result, patients are generally separated into heterogenous pools of high-risk versus low-risk at the time of a newly-diagnosed malignancy or following HSCT [15]. This approach often falls short in the “real-life” management of patients who receive multiple lines of chemotherapy with relapsed malignancy, genetic or occupational predisposition for mold infections, a prior history of mold disease, or receive novel biological agents or immunosuppressive chemotherapy. Therefore, a goal for any prognostic model is to provide a practical approach for improving the precision and accuracy of risk prediction for all patients so clinicians can make better informed decisions. Before discussing specific risk models, however, it is important to briefly review the major risk factors that predispose patients with hematological malignancies to developing an IMD.

### 2.1. Underlying Malignancy and Status

Acute myeloid leukemia (AML) and myelodysplastic syndromes (MDS) have historically been associated with the highest reported rates of IMD, ranging from 5–25% [4,16,17]. The period at greatest risk for IMD is typically during initial induction chemotherapy, which typically results in prolonged (>21 days) and profound (<500 PMN/mm^3^) neutropenia [18]. Other factors in AML patients that may be associated with higher rates of IMD include older age, poor prognosis for achieving complete remission (CR) due to unfavorable cytogenetics or relapsed malignancy, and presence of multiple baseline comorbidities or poor performance status [4]. Patients who receive induction chemotherapy in non-HEPA filtered rooms, or an occupational history of heavy spore exposure (e.g., farming, construction) may also be at higher risk. Relapse of IMD is also a concern in any patient undergoing consolidation chemotherapy with a previous history of fungal infection during their induction regimen [19]. Survival rates among patients with AML have improved in recent years despite relatively few changes in frontline chemotherapy protocols due in part to improved supportive care measures and the introduction of more effective prophylaxis options such as posaconazole [20].

Older patients with transformed MDS are at high risk for IMD if they receive AML-like treatment regimens. However, hypomethylating agents such as azacytidine or decitabine are increasingly being used in place of cytosine arabinoside (ARA-C), resulting in lower rates of infection complications, including a probable or proven IMD of 2–5% [21,22,23].

Adult patients with acute lymphoblastic leukemia (ALL) are generally considered to be at moderate risk for developing IMD, with most case series having reported rates of probable or proven IMD between 2–5% [17]. A recent randomized study comparing prophylaxis with liposomal amphotericin B to placebo in adult patients with ALL reported rates of probable IMD of 7.5% and 9%, respectively [24]. The relatively higher incidence of probable IMD observed in this study may have reflected recent trends in the use of more intensive chemotherapy regimens in adults designed to improve long-term survival, which are associated with higher rates of infection during induction chemotherapy [25]. The risk of IMD may also be increased in patients with relapsed ALL, especially in patients who receive regimens with high doses of dexamethasone [26]. In contrast, patients with Philadelphia-positive ALL receiving treatment with tyrosine kinase inhibitors (imatinib, dasatinib, nilotinib, and bosutinib) as part of standard or reduced-intensity regimens appear to have a lower risk of developing an IMD (3–5% incidence) [27,28,29].

Patients with chronic lymphoproliferative disorders such as non-Hodgkin’s and Hodgkin’s lymphoma, chronic myelogenous leukemia, or multiple myeloma are generally considered to be at low risk for IMD (<2% incidence). However, select subsets of patients with extensively-treated lymphoma who receive intensive chemotherapy regimens of high-dose corticosteroids followed by autologous HSCT often experience prolonged neutropenia and with rates of IMD like patients with AML/MDS undergoing induction chemotherapy [30]. Patients who receive intensive chemotherapy regimens for CNS lymphomas in combination with drugs that target B-lymphocyte pathways, particularly the Bruton’s tyrosine kinase inhibitor ibrutinib, may be at especially high risk for cryptococcosis, pneumocystis pneumonia, and aspergillosis involving the central nervous system [31]. In one case series the use of ibrutinib in combination with temozolomide, etoposide, cytarabine and liposomal doxorubicin, rituximab and dexamethasone was associated with rates of invasive aspergillosis of 44% [30,31,32]. In contrast, the use of ibrutinib monotherapy in patients with chronic lymphocytic leukemia was associated with low rates of invasive fungal disease (0.5–1.6%) that were comparable to treatment with alkylating agents or monoclonal antibodies, which are no longer considered to be the main standard of care [32,33].

Relatively few data are available describing the risk of IMD among patients with myeloproliferative neoplasms including chronic myelogenous leukemia, polycythemia vera, essential thrombocytopenia, and myelofibrosis [4]. Accurate estimates of IMD risk in these populations are confounded by the increasing use of tyrosine kinase inhibitors (imatinib, dasatinib, nilotinib, bosutinib, and ponatinib) for targeting the BCR-ABL oncoprotein in chronic myelogenous leukemia, which leads to disease control in most patients. Similarly, targeted therapy with JAK (Janus Kinase) inhibitors in patients with myelofibrosis appears to be associated with a low risk of IMD [34,35].

Severe aplastic anemia is characterized by a reduction in the production of hematopoietic progenitor cells resulting in severe pancytopenia [36]. Infection is a major cause of death and is directly related to prolonged neutropenia. The risk of fungal infection is further increased by treatments that reduce T-cells (anti-thymocyte globulin) or function (corticosteroids, calcineurin inhibitors), however *Candida* spp. appear to be much more common than invasive molds [37].

### 2.2. Conditions Associated with Disease Treatment

Autopsy studies performed in the early 1960s identified prolonged neutropenia as a major predisposing risk factor for IMD [38,39]. Gerson and colleagues [40] later demonstrated that neutropenia persisting longer than three weeks was the most important risk factor for development of invasive pulmonary aspergillosis in patients with acute leukemia. During the first two weeks of neutropenia, patients developed signs of invasive risk of invasive pulmonary aspergillosis at a rate of approximately 1% per day that increased to 4.3% per day between the 24th and 36th days of neutropenia. Of the 13 patients who remained neutropenic at 28 days, 7 (54%) had developed signs of invasive pulmonary aspergillosis.

The European Organization for Research and Treatment of Cancer /Invasive Fungal Infections Cooperative Group and the National Institute of Allergy and Infectious Diseases Mycoses Study Group (EORTC/MSG) Consensus Group have defined neutropenia as a host factor for invasive fungal disease when it presents as <500 cells/mm^3^ for >10 days [41]. Typically, the median duration of neutropenia prior to the first signs of IPA ranges from 16–25 days, but for some patients with multiple risk factors, disease onset may occur before 10 days [3].

Lymphopenia (<300 cells/mm^3^) and monocytopenia (<10 cells/mm^3^) are infrequently the sole predisposing risk factors for IMD, but often indicate delayed immune reconstitution after chemotherapy or allogeneic HSCT [4,42]. In an analysis of 1248 allogeneic HSCT recipients, neutropenia (HR 2.2; 1.3–3.6, *p* < 0.01), lymphocytopenia (1.4, 1–2; *p* = 0.05), and monocytopenia (HR 1.8, 1.7–3.4, *p* < 0.01) were independently associated with increased risk of mold disease within one year of transplantation [43]. Mikulska et al. identified the presence and duration of lymphopenia as independent risk factors for early mortality from invasive aspergillosis in recipients of allogeneic HSCT from alternative donors [42]. Lewis et al. reported that patients with pulmonary mucormycosis who presented with lymphocyte counts <100 cells/mm^3^ had a 4-fold higher rate of mortality versus patients without lymphocytopenia [44].

Glucocorticoid therapy at supraphysiologic doses have long been associated with the development of mold infections [45], but the cumulative dose that places a patient at increased risk varies from study to study and often depends on concomitant chemotherapy, type of allogeneic HSCT and severity of GVHD [46]. The EORTC/MSG consensus definitions for invasive fungal disease include corticosteroids as a host factor for mold disease when a patient has received a mean minimum dose of 0.3 mg/kg/day prednisone equivalent for greater than 3 weeks [41]. Notably, the EORTC/MSG criteria do not consider inhaled corticosteroids to be a host factor even though cases of invasive pulmonary aspergillosis have been described in critically-ill patients with chronic-obstructive pulmonary disease [47,48].

In patients who undergo allogeneic HSCT, high cumulative doses of glucocorticoids are the most frequently identified risk factor associated with invasive aspergillosis after engraftment [49,50,51,52]. O’Donnell and colleagues reported that use of high-dose prednisone (0.5–1.0 mg/kg per day) for graft-versus-host-disease increased the risk six-fold versus lower dose prednisone regimens (0.25 mg/kg/day) for developing invasive aspergillosis [53]. Similarly, Marr and colleagues reported that glucocorticoid doses of 1.9 mg/kg per day, 1.9–3.0 mg/kg per day, and greater than 3 mg/kg per day were associated with IMD risks of 5%, 10%, and 14%, respectively [49]. Ribaud and co-workers reported that the 60-day risk of death increased from 12% to 80% if allo-HSCT recipients had received a cumulative prednisolone dose of greater than 7 mg/kg in the week preceding diagnosis of IMD [51].

### 2.3. Hematopoietic Stem Cell Transplantation

Allogeneic HSCT recipients have among the highest reported incidence of IMD in studies ranging between 7 and 15% [4]. The risk period is bimodal, with early risk for disease (first 40 days) associated with prolonged neutropenia prior to stem cell engraftment, and later invasive mold disease (often after day +70 or +100) associated with immunosuppressive therapy for controlling GVHD. Several additional pre- and post-transplant factors have been reported to influence IMD risk. Pre-transplant risk factors include the type of transplant (matched-related donor, umbilical-cord donor, or haploidentical/mismatched donor) [43,54,55] stem cell dose [56], receipt of T-cell depleting agents (e.g., anti-thymocyte globulin or alemtuzumab) or a T-cell depleted graft [43,57], polymorphisms in donor genes important for detection of fungal antigens and antifungal innate immune responses (i.e., Toll-like receptor 4, Dectin-1, and Pentraxin-3) [58,59,60], and iron overload associated with frequent transfusions [43,61,62].

Post-allogeneic HSCT risk factors for IMD include the rate of immune reconstitution (time to engraftment and recovery from neutropenia, lymphocytopenia, and monocytopenia), recovery of natural-killer cell populations [63], development of acute graft versus host disease and its treatment with immunosuppressive agents (corticosteroids, anti-T cell therapies), and development/reactivation of viral co-infections, particularly cytomegalovirus (CMV) [43].

The incidence of IMD following autologous HSCT is lower, with reported incidence ranging between 3–8% [64,65,66]. The incidence is influenced by the underlying malignancy, number and types of chemotherapy cycles prior to transplantation, previous history of IMD, as well as antifungal prophylaxis [4].

### 2.4. Patient Comorbidities

Besides older age, several underlying comorbidities or poor performance status overall are associated with increased susceptibility to IMD. Patients with uncontrolled diabetes mellitus may exhibit impaired neutrophil migration and fungal cell phagocytosis and T-cell dysfunction [67]. In the setting of metabolic acidosis, uncoupling of free iron from carrier proteins in blood enhances fungal growth and is an important risk factor for disseminated mucormycosis [68,69]. Smoking and chronic pulmonary disease were similarly identified as pre-chemotherapy risk factors for the development of IMD during initial remission-induction therapy [16]. Poor nutritional status or cachexia associated with advanced disease, often manifesting as hypoalbuminemia, was associated with 3-fold lower odds of developing IMD following allogeneic HSCT for each gram/deciliter increase in serum albumin [70].

Viral co-infections, particularly cytomegalovirus (CMV) and influenzae are associated with increased risk of invasive pulmonary aspergillosis. CMV viremia and recipient CMV serostatus have been identified as risk factors following allogeneic HSCT for both early and late-onset IMD, which may be enhanced in patients who developed prolonged neutropenia following treatment with ganciclovir [43,71,72]. However, it is still debated whether CMV replication directly impairs antifungal immunity or is a signal of already impaired cellular immunity that allows permissive growth of molds [73,74]. Nevertheless, the clear temporal association of CMV viremia or infection with the development of IMD suggests CMV replication or disease can be a powerful predictive factor for mold disease [71].

The link between severe influenzae and invasive aspergillosis has been increasingly described in both non-immunocompromised and immunocompromised hosts [75,76,77,78,79]. It has been hypothesized that the evolution of more virulent influenza strains, as exemplified by the pandemic H1N1 strain, are associated with more severe lymphopenia and diffuse damage to the respiratory mucosa during infection that predispose hosts to fungal invasion [79,80]. Influenza preceding IMD is associated with high rates of respiratory failure and mortality, highlighting the importance of prompt diagnosis and initiation of antifungal therapy.

### 2.5. Environmental and Occupational Risk Factors

Repeated exposures to high fungal spore counts associated with farming, construction work, gardening or composting, and perhaps geoclimatic factors increase colonization and persistence of fungal spores in the respiratory tract and place the patient at increased risk for developing IMD during induction chemotherapy [16,81,82]. Outbreaks of invasive mold disease have been repeatedly described following hospital construction and renovation that results in dust contamination and dispersal of fungal spores [83]. Similarly, some studies have documented a relationship between environmental contamination by *Aspergillus* and other fungal species and the incidence of invasive aspergillosis [84]. This risk may be increased if patients are admitted for intensive chemotherapy or transplantation to rooms without positive pressure high efficiency particulate air (HEPA) filtration [85].

## 3. Risk Models for Invasive Mold Disease

### 3.1. Neutropenia-Associated Risk Measured by the D-Index

**Figure 1:**
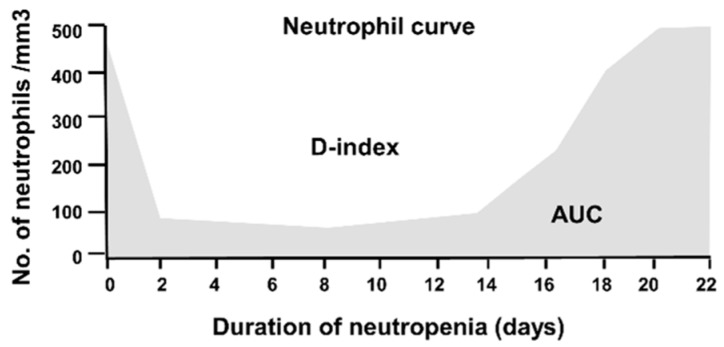
D-index area over the neutrophil curve: AUC areas under the neutrophil curve.

Given the high frequency and importance of neutropenia as a key risk factor for IMD, several investigators have focused on the development of tools that measure both the intensity and duration of neutropenia to predict risk of IMD. Portugal et al. proposed an index (D-index) to improve the assessment of risk for IMD in neutropenic patients. The D-index represents the difference in the area under the curve (AUC) for neutrophil counts over time versus an area resulting from a normal neutrophil count. This difference is geometrically represented as the area over the neutrophil curve (Figure 1) [86]. The investigators also evaluated the prognostic performance of the cumulative D-index score (c-D-Index), which represents the D-index from the start of neutropenia until the date of the first clinical manifestation of IMD. Compared to just measuring the duration of neutropenia, the D-index and c-D-index better discriminated patients (*n* = 11) who developed IMD with area under the receiver operator curve (aROC) values of 0.86 and 0.81 versus patients who did not develop an IMD (*n* = 33). At a cut-off of 6200, the sensitivity and specificity of the D-index was 100% and 58%, respectively and for the c-D-index value of 5800 the sensitivity and specificity were 91% and 58% respectively. Over an incidence range of 5–15%, the positive predictive value (PPV) of the D-index was relatively low (11–30%) but the negative predictive value was high (97–99%) suggesting that the D-index may be useful for “screening-out” lower risk patients with neutropenia who are unlikely to develop a mold infection [86].

In a follow-up prospective study among 29 patients with acute leukemia undergoing remission-chemotherapy, the investigators utilized the D-index and galactomannan screening to stratify patients as low (<3000), intermediate (3000–5800), and high risk (>5800) for IMD [87]. Although a positive galactomannan result or clinical symptoms triggered a diagnostic workup in similar numbers of patients irrespective of the D-index risk stratification (58–73%), patients in the low risk D-index group were less likely to receive antifungal therapy (17% vs. 54–67%) and no cases of IMD were diagnosed in the low risk group versus 67% and 45% of the patients classified as high and moderate risk, respectively.

### 3.2. A risk score for Predicting IMD in Lymphoma Patients Receiving Salvage Chemotherapy

Takaoka et al. colleagues retrospectively analyzed 177 consecutive patients who received salvage chemotherapy for active lymphoma (705 courses in total) [88]. The IMD incidence rate was 2.3% (6 probable and 6 possible cases). Multivariate analysis revealed that relapsed refractory disease, receipt of two or more treatment courses, and neutropenia (ANC < 500 cells/mm^3^) were independently risk factors associated with the development of IMD. Using these variables, the authors developed a simple weighted risk score: 1 point for refractory therapy, 1 point for two or more treatment lines, 2 points for three or more treatment lines, and 1 point for neutropenia. By applying the score, the authors were able to differentiate a subgroup of lymphoma patients with a higher incidence of IMD by day 80 if the score was above 3 (9% incidence) versus below 2 (0.19% incidence). However, given the retrospective nature of the analysis, it is unclear at what time the score could be applied to predict future IMD. Additionally, the inclusion of EORTC/MSG possible cases of IMD might be questioned as potentially more than half of these cases did not have IMD, especially in the setting of relapsing lymphoma which can produce nodular consolidations in the lung indistinguishable from lymphoma by standard chest CT [89].

### 3.3. A Risk Score for Predicting IMD Risk Post-Engraftment in Adult Allogeneic HSCT Recipients

Montesinos et al. [90] analyzed risk factors for probable or proven IMD among 404 allogeneic HSCT recipients who engrafted and survived more than 40 days after transplant. The one-year cumulative incidence of IMD in their study cohort was 11%. Five risk factors identified in multivariable analysis (age greater than 40 years, more than one previous HSCT, pre-engraftment neutropenia lasting more than 10 days, extensive and chronic GVHD, and CMV reactivation) were used to construct as risk score for stratifying patients into low risk (0–1 factor, cumulative incidence 0.7%) intermediate risk (2 factors, cumulative incidence 9.9%), and high-risk (3–5 factors, cumulative incidence 24.7%) categories. Although the authors suggest the score could be used to assess risk at patient discharge, two of the five risk factors (extensive chronic GVHD, CMV reactivation) would likely develop later after discharge, therefore patients would need to be reassessed periodically to fully apply the risk score.

### 3.4. Predicting Invasive Fungal Infection in Pediatric Allogeneic HSCT Recipients

Hol and colleagues retrospectively analyzed pre- and post-transplant predictors of invasive fungal disease among 209 pediatric recipients of allogeneic HSCT with at cumulative incidence of IMD of 12% (mostly molds) [91]. Patients were classified as high or low risk based on pre-transplant risk factors that included: age < 10 years, gender, treatment-related mortality risk predicted by the EBMT risk score, diagnosis type, use of mold-active prophylaxis, prior history of invasive fungal infections, donor type, donor relation and match, conditioning regimen, transplant number, and presence of galactomannan in pre-transplant bronchial alveolar lavage samples. Post-transplant risk factors included duration of neutropenia, aGVHD > grade II, extensive cGVHD, and high-dose corticosteroids (>1 mg/kg/day for at least one week). In multivariate analysis, an EMBT score predicted that treatment related mortality risk >20% was the only factor associated with the occurrence of an IFI, while posttransplant high-dose steroids were the only predictor of invasive fungal infection. After adjustment for pre-transplant treatment related mortality risk and use of corticosteroids, the odds of survival were significantly lower in children who developed IMD versus those who did not (OR 0.30, 95% CI 0.13–0.71, *p* = 0.006).

### 3.5. Applying Comorbidity Index to Predict IMD after Allogeneic HSCT

Prognostic risk models are frequently used to predict non-relapse mortality among patients undergoing allogeneic HSCT. In 2005, Sorror et al. [92] introduced the hematopoietic stem cell transplantation-comorbidity index (HSCT-CI) assessed prior to transplantation as a means of predicting non-relapse mortality, that was later updated with additional clinical risk factors [93]. Busca et al. recently explored whether the HSCT-CI could have similar utility in predicting the risk for adult patients undergoing allogeneic HSCT for developing fatal IMD [94]. Among 360 retrospectively-analyzed patients who underwent allogeneic HSCT, 8.5% of patients developed EORTC/MSG probable or proven IMD that was significantly higher among patients with and HSCT-CI score of ≥3 (12%) compared to patients with HSCT-CI scores of 0–2 (5%). Pulmonary comorbidities were the most common pre-transplant risk factor associated with the development of IMD. Advanced disease at the time of transplant, acute grade II-IV GVHD, and a comorbidity score ≥ 3 were independent risk factors for non-relapse mortality.

### 3.6. Development of a Universal IMD Risk Model for Patients with Hematological Malignancies

**Figure 2:**
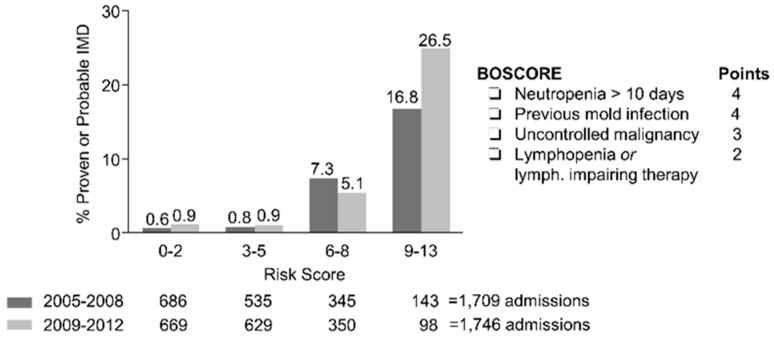
BOSCORE distribution versus observed 90-day cumulative incidence of IMD in the retrospective (2005–2008) development and prospective (2009–2012) validation cohorts.

Many decisions regarding the management of IMD (e.g., galactomannan screening, antifungal prophylaxis computer tomography in symptomatic febrile patients) are made at the time or soon after admission to the hospital. With this idea in mind, we sought to develop a universal prognostic risk model that could be assessed at the time of each hospital admission to predict an individual patient’s risk for developing IMD in the future [15]. Seventeen risk factors assessed at the time of patient admission were first retrospectively analyzed using a data registry of 840 patients with hematological malignancies over 1709 admission episodes lasting more than 5 days from 2005–2008. The data registry was maintained by a dedicated data manager and adjudicated periodically by an attending hematologist with expertise in infectious diseases. Although a total of 11/17 analyzed risk factors correlated with IMD in univariate analysis, only four risk factors were retained in the final multivariable model that were predictive of probable or proven IMD within 90 days of admission: (i) active malignancy (not in remission); (ii) PMN < 500 cells/mm^3^ > 10 days or projected prolonged with chemotherapy; (iii) severe lymphocytopenia < 50 cells/mm^3^, or lymphocyte-impairing therapies such as calcineurin inhibitors; (iv) and prior history of IMD. These variables were then used to construct a weighted risk score (BOSCORE) with a scale of 0–13. A risk score threshold of < 6 differentiated patients with low (<1%) versus higher (>5%) probability thresholds of IMD. The score was then prospectively validated in our institution in 855 patients over 1746 admissions from 2009–2012 (Figure 2). The discrimination and calibration of the score for predicting IMD were similar in the validation cohort of patients despite introduction of routine posaconazole prophylaxis in AML/MDS patients undergoing induction chemotherapy, with an aROC of 0.84 (95% CI 0.79–0.89) and negative predictive value of 0.99 (95% CI 0.98–0.99) at a 5% predicted probability threshold cut-off.

**Figure 3:**
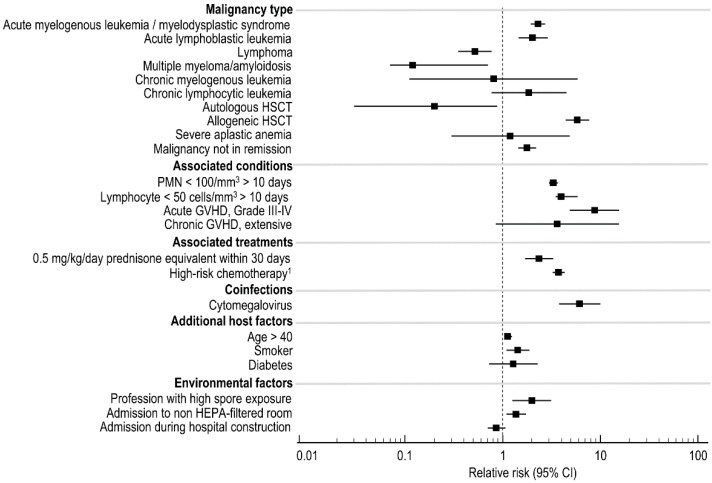
Risk factors in univariate analysis associated with the development of EORTC/MSG defined probable or proven invasive mold disease (*n* = 133) among 1944 adult inpatients (*n* = 4127 admissions) undergoing treatment for a hematological malignancy from 2007–2016 at the Seràgnoli Hematology Institute in Bologna, Italy.

We recently recalibrated the BOSCORE with additional risk factors to predict the 60-day probability of developing probable or proven IMD using 1944 patients with 4127 admissions from 2007–2016. The overall incidence of probable or proven IMD was 3.3%. Most of the analyzed risk factors were associated with the development of IMD (Figure 3), however only seven risk factors were retained in the final multivariable model: (i) prior history of IMD, (iii) receipt of 0.5 mg/kg prednisone equivalent within 30 days, (iii) uncontrolled malignancy; (iv) receipt of high-risk chemotherapy—e.g., any conditioning for allogeneic HSCT, high-dose ARA-C, fludarabine, and idarubicin (FLAI), or ifosfamide, carboplatin, etoposide (ICE); (v) PMN < 100 cells/mm^3^ for >10 days or anticipated prolonged neutropenia; (vi) total lymphocyte count <50 cells/mm^3^; and (vii) CMV reactivation (DNA > 1000 IU/mL in serum) or disease.

**Figure 4:**
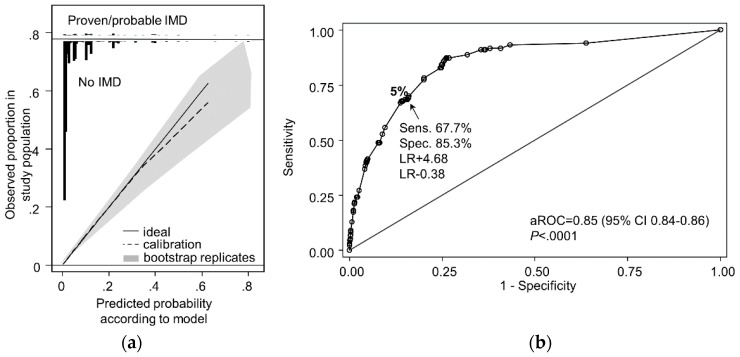
Revised BOSCORE model calibration and discrimination. Panel (**a**) shows the relationship between a perfectly calibrated model (solid line) and the observed incidence of EORTC/MSG proven, or probable mold disease fitted using the Loess smoothing algorithm (dashed line). Black bars on the top frame of the graph show the relative risk distribution of admissions without IMD (downward pointing spikes) or patients with proven or probable IMD (upward pointing spikes). The gray shaded area shows observed versus predicted incidence of proven or probable mold disease in 100 bootstrapped resampled datasets with varying IMD incidence of 2–9%. Panel (**b**) shows the area under the receiver operator curve (aROC) and predicted performance for EORTC/MSG probable or proven IMD at a prediction cut-off of 5%. Sens., sensitivity, spec. specificity, LR, likelihood ratio.

The re-calibrated multivariate risk model displayed good calibration and discrimination in both low and higher risk groups and provided consistent predictions during internal validation with bootstrap resampled populations over an IMD incidence range from 2–9% (Figure 4a). When a 5% threshold was applied to differentiate low versus high-risk patients, the risk model correctly identified 60-day IMD outcomes in 85% of patients (Figure 4b). Among patients who developed IMD but were predicted to be low risk at admission, most (74%) fell just below the provisional 5% threshold (i.e., 3.5%–4.9% risk).

**Figure 5:**
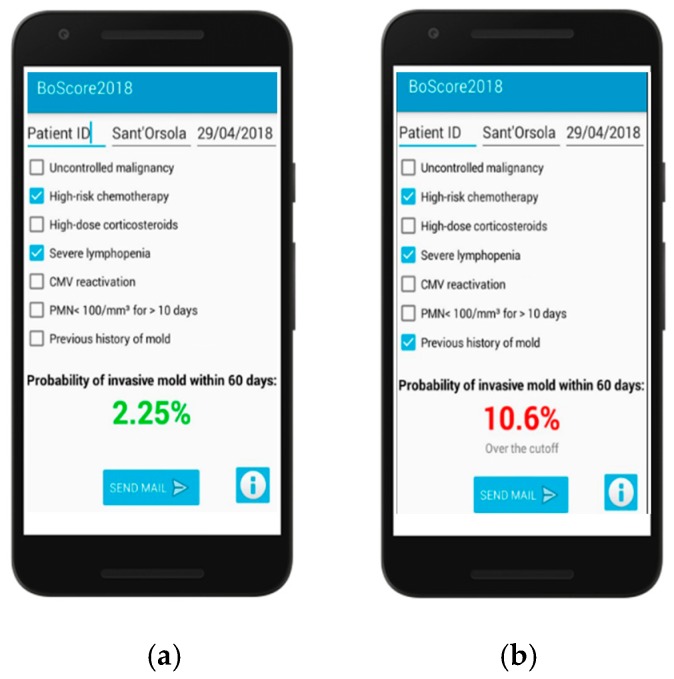
Smartphone-assisted prognostic model assessments of lower-risk (**a**) and higher-risk (**b**) nonneutropenic patient. The probability of IMD within 60 days is estimated using the formula: Risk = (0.68 × uncontrolled malignancy) + (0.79 × high-risk chemotherapy) + (0.80 × high-dose corticosteroids) + (0.89 × severe lymphopenia) + (1.14 × CMV reactivation) + (1.52 × prolonged neutropenia) + (1.64 × previous mold disease) − 5.45. To calculate the 60-day probability from the formula, the calculated result is first converted from log odds to odds (eRisk); then odds must be converted to probability using the formulae: Risk/(1 + Risk).

The risk model was subsequently developed as a smartphone application (Figure 5) that requires users to only check boxes of each risk factor present on admission and the 60-day risk for IMD is automatically calculated with links to recommendations institutional diagnostic and treatment algorithms based on the patient’s estimated risk.

**Figure 6:**
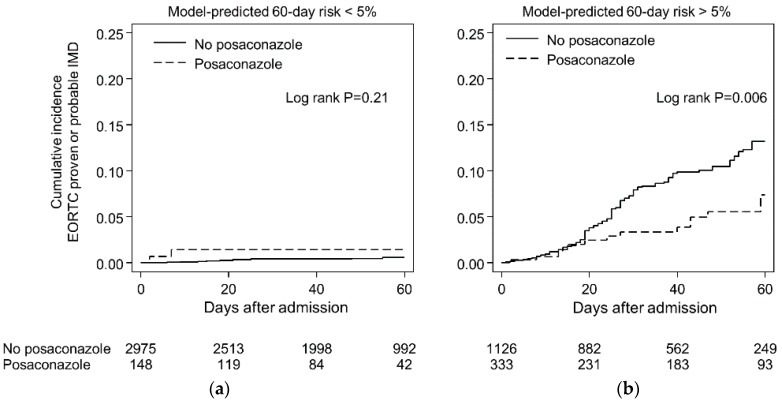
Impact of posaconazole prophylaxis on the 60-day cumulative incidence of IMD in patients with model predicted risk of IMD <5% (**a**) and >5% (**b**). Note the nonsignificant higher rate of IMD in low risk patients (Panel A) is explained by two patients. One patient had a predicted risk at the risk cut-off (0.5) and prior IMD as the only risk factor (receiving posaconazole secondary prophylaxis). He was diagnosed with breakthrough IMD within 7 days of admission with fever. The second patient receiving posaconazole prophylaxis was admitted for treatment of extensive chronic graft versus host disease (he was not receiving corticosteroids) and only had severe lymphopenia at the time of admission. His predicted risk was 2.5%. During his work-up he had no fever but a serum galactomannan was positive and a CT following admission was consistent with IMD.

The true test of any prognostic risk model is its acceptance by practitioners and effects on clinical decision making [95]. The high negative-predictive value of the BOSCORE (0.96–1.0) across a wide range of patient groups with varying incidence of IMD (0.4–8%) is used by clinicians in our institute to screen out the majority (> 67%) of patients admitted who are unlikely to benefit from intensive monitoring of serum galactomannan or posaconazole prophylaxis (Figure 6). Indeed, routine serum testing of galactomannan in serum of patients with a low pretest probability of IMD (i.e., <2%) may result in more frequent false-positive rather than true-positive results resulting in potentially unnecessary invasive diagnostic procedures and antifungal therapy [96]. Patients at greater than 5% risk are considered for more intensive management by a diagnostic-driven algorithm. This algorithm consists of twice weekly serum galactomannan screening plus immediate low-dose CT (within 24–48 h of fever or signs of infection) followed by CT pulmonary angiography ± bronchoscopy with culture or possible biopsy if the patient has evaluable lesions and can tolerate the procedures [97].

Very-high risk patients (>10%) may be considered eligible for posaconazole prophylaxis, even if they do not have AML/MDS or have undergone allogeneic HSCT. For example, we routinely use the risk model to help identify patients with acute leukemia undergoing consolidation chemotherapy who require mold-active antifungal prophylaxis. We have also found that that the risk model identifies small subsets of patients with lymphoma (6%) and myeloma (3.4%) who have equivalent risk for developing IMD as an AML patient undergoing remission/induction chemotherapy. These unique subsets of patients are then targeted for more intensive diagnostic intervention.

## 4. Future Perspectives

Because accurate risk assessment is fundamental to the effective management of mold infections and stewardship of diagnostic and treatment resources, it is likely that interest in prognostic risk models will increase. However, adherence to several fundamental principles during model development and reporting should be encouraged. First and foremost, there should be a clear explanation of the patient population used to develop the predictive model and the timing of assessment- i.e., when IMD is already present (diagnostic model) or to predict the risk of IMD in the future (prognostic model). Steps involved in the development, analysis and validation of the model should be clearly reported according to standards described in the Transparent Reporting of a Multivariable Prediction Model for Individual Prognosis Or Diagnosis (TRIPOD) Guidelines [98]. Adherence to these guidelines improves the clarity in communication and allows researchers from other institutions to better assess the generalizability and risk of bias of published models [98].

Validation studies are often considered to be the benchmark of whether a developed model is of high quality or clinically useful. The real aim of model validation, however, is to measure the model’s predictive performance in either resampled participant data of the development data set (often referred to as internal validation) or in other, independent participant data that were not used for developing the model (often referred to as external validation) [98,99]. In this respect, the key difference is the generalizability of the performance characteristics of the model, which should ideally be confirmed before the model is used in clinical care like any new diagnostic test [95]. Prediction models developed in one institution may not necessarily be equally useful in another setting, as illustrated with recent studies of *Candida* prediction models in non-neutropenic patients [8]. Indeed, it is not uncommon that existing models must be adapted to local circumstances or with new predictors before application in a local setting. This adaption will be impossible if local hospitals do not have surveillance systems in place where these infections are managed. Finally, research on the uptake and effect of prognostic models on clinical decision-making and patients’ outcomes (impact studies) are needed to confirm the usefulness of the prognostic model in clinical care.

Outside of daily clinical use, Harrell and colleagues have identified several areas of clinical research where prognostic risk models are applicable [10]. Risk models are useful in the evaluation of new diagnostic technologies, as estimates derived both with and without the new test can be compared to measure the incremental prognostic information provided. White, Parr and Barnes recently used this approach to evaluate how genetic risk factors with early diagnostic markers can improve predictions of which neutropenic patients with hematological malignancies will develop invasive aspergillosis [100]. The investigators found that compared to clinical risk factors alone (allogeneic HSCT, respiratory virus infection), the incorporation of genetic risk factors (DECTIN-1, DC-SIGN mutations) plus a positive PCR result increased the predicted probability of invasive aspergillosis from 7% to 56.7%. This combined prognostic-diagnostic model approach was associated with good discrimination (aROC 0.86) and could substantially reduce the percentage of patients administered preemptive therapy to only 8.4% of the population of patients with febrile neutropenia.

A second means by which a validated prognostic model could benefit clinical research is to enable researchers to better estimate the effect of a single factor (e.g., antifungal prophylaxis) on patients’ outcomes in observational data where many uncontrolled confounding factors also affect risk.

Finally, prognostic models can improve the design and enrollment of clinical trials for invasive mold diseases. Both the decision concerning which patients to randomize and the design of the randomization process (for example, stratified randomization using prognostic factors) are improved by more accurate prediction of patient risk before randomization. Accurate prognostic models can be used to test for differential therapeutic benefit or to estimate the clinical benefit for an individual patient in a clinical trial, because low-risk patients are likely to have less absolute benefit. These areas are largely unexplored for prognostic models and clinical studies of IMD.

## 5. Conclusions

Compared to therapeutic trials and etiological research, prognostic risk models for IMD have received relatively little attention. However, several studies have now shown that development of such models can improve the accuracy of risk prediction, and incorporation of these models into the clinical management of patients has the potential to improve diagnostic and antifungal stewardship, as well as personalized treatment of invasive mold diseases in patients with hematological malignancies.
